# Legal Preemption and the Prevention of Chronic Conditions

**DOI:** 10.5888/pcd13.160121

**Published:** 2016-06-30

**Authors:** James G. Hodge, Alicia Corbett

**Affiliations:** Author Affiliation: Alicia Corbett, Public Health Law and Policy Program, Sandra Day O’Connor College of Law, Arizona State University, Tempe, Arizona.

## Abstract

State and local legal innovations to address chronic conditions are an ongoing source of public health improvements. For decades, some of the most ingenious law and policy ideas to address the underlying causes of chronic conditions and their contributing factors have emerged from state or local public sector grassroots initiatives in diverse areas, including tobacco use, safe housing and transportation, and environmental hazards. These reforms, however, are susceptible to invalidation through the legal doctrine of preemption. Embedded throughout our constitutional system, preemption refers to how state or local laws may be averted, displaced, or negated by conflicting laws at a higher level of government. Preemption can be complex in concept and application, leading to considerable confusion among public health leaders seeking to generate meaningful policy proposals. The objective of this article is to unravel the legal concept of preemption, explain its use as a tool to both thwart or further public health interventions, and offer practical guidance for how to legally navigate around it to address factors underlying chronic conditions.

## Introduction

State and local legal innovations to address chronic conditions are an ongoing source of public health improvements. For decades, some of the most ingenious law and policy ideas to address underlying causes of, or contributing factors to, chronic conditions have emerged from grassroots initiatives (1). Whether developed locally or via state governments, legal advances have stimulated public health reforms in diverse areas, including tobacco use, safe housing and transportation, and environmental hazards. These reforms, however, are susceptible to invalidation through preemption, a legal doctrine embedded in our constitutional system, which refers to how a state or local law may be averted, displaced, or negated by conflicting laws at a higher level of government. Thus, federal laws can preempt state or local laws, and state laws can preempt local laws. 

Though easily stated, preemption is complex as applied, leading to confusion and angst among public health leaders seeking to generate meaningful policy proposals. In this article, we unravel the legal concept of preemption, explain its use as a tool to both thwart or further public health interventions, and then offer practical guidance for how to navigate around it to legally address factors underlying chronic conditions.

## A Brief Primer on Legal Preemption

The premise that a higher level of government may override the laws of a lower level of government is simple in theory but complicated as applied. Understanding preemption starts with the US Constitution. It grants the federal government limited, defined powers (eg, to regulate interstate commerce, to tax, and to set conditions on the receipt of federal funds). By design, federal legislators, officials, and judges are constrained to act pursuant to the powers specifically granted to them by the Constitution and federal law. Over time these powers have expanded to allow federal intrusions into public health areas unforeseen by the original framers of the Constitution. Still, as long as federal laws are constitutionally authorized, they prevail over any conflicting state or local statutes, regulations, judicial decisions, or other laws (2).

This same basic constitutional scheme also plays out at the state level. State legislation, regulations, and judicial decisions preempt conflicting local laws. One key distinction between federal and state preemption extends from the actual sources of government powers. Although principles of federalism restrain the federal government from directing state governments, the same is not true for state control of local governments. As sovereign governments, states allot powers to counties, cities, or other municipalities. Whatever powers a state doles out to localities can generally be withdrawn or tied to other stringent conditions that impact local authority, sometimes known as “home rule,” unless states constitutionally guarantee municipalities a level of authority that cannot be later taken away.

In summary, federal laws are supreme and can override conflicting state or local laws. State laws cannot preempt federal laws, but they can negate conflicting local laws (such as a local ordinance prohibiting public health practices that state law requires). If only preemption was this easy in application. Unfortunately, it is not. At the federal level, Congress can expressly (via specific legislative language) or impliedly (based on Congressional intent as interpreted by courts) preempt state or local laws completely, partially, or not at all. Federal legislation may establish national, uniform standards that state or local laws cannot contravene directly in 3 primary ways.

First, state or local laws imposing stricter standards than those at the federal level can be preempted if the federal standard is meant to act as a “ceiling,” which measures taken at lower levels of governments cannot exceed. Second, state or local laws that provide less restrictive standards may also be preempted if meeting a lower standard defeats the purpose of federal law. In this way, sometimes federal actors create a legal “floor,” which state or local laws cannot go below but can surpass through greater requirements (3). Third, sometimes federal law “occupies a field” so completely as to preempt similar state or local laws (even if federal law insufficiently achieves essential public health outcomes). In this way, preemption can thwart laudable interventions if federal law strips state or local governments of their ability to address public health issues (4). State legislatures can preempt local laws in similar ways. Correspondingly, local public health exercises cannot extend beyond limited jurisdictional boundaries or conflict with or impair federal or state law.

## Preemption and Prevention of Chronic Conditions

Preemption has the potential to impede innovative legal public health approaches. Several states, for example, legislatively prohibit all local regulation of toys provided with children’s meals at restaurants, but offer no alternative state-based regulations (5). Conversely, preemption can be used to promote healthy behaviors through greater legal uniformity. In 1990, for example, Congress banned smoking on nearly all domestic flights, snuffing out varying state-based restrictions by aligning public health and industry interests to reduce tobacco-related chronic conditions among airline employees and passengers (6).

Of course, industry interests do not always comport with public health objectives. To create greater business efficiencies across state boundaries, industries may seek uniformity of regulatory requirements. Industry groups or others may lobby for the inclusion of preemptive language in federal or state legislation and then argue how public health laws or efforts at lower levels of government are overridden (7). Examples are abundant. Federal statutes preempt some state lawsuits alleging faulty products against medical device manufacturers as well as cases against manufacturers of automobiles without airbags (8). California enacted a uniform state law in 2008 to preempt local laws requiring restaurants to post nutritional information (9). Most states legislatively prevent local governments from imposing stricter restrictions on pesticide use (10) or regulating firearms to some extent or at all (11). In 2001, Ohio’s state legislature preempted Cleveland’s ordinance prohibiting liquor advertising (12).

State or local laws that conflict with federal laws on the manufacture, sale, and advertising of tobacco are frequent targets of preemption litigation (1). Tobacco manufacturers successfully argued against a 1999 Massachusetts state regulation restricting outdoor and point-of-sale cigarette advertising (13) and a 2009 New York City law requiring graphic tobacco warning signs in retail tobacco sales areas (14). Courts deemed both laws as preempted by the Federal Cigarette Labeling and Advertising Act (15), which the US Supreme Court stated is designed to “protect the national economy from interference due to diverse, non-uniform, and confusing . . . regulations . . . ” (13). Even non-tobacco legislation may hinder public health goals. Maine’s tobacco delivery act requiring tobacco shippers to verify a buyer’s legal age was preempted in 2008 by federal legislation governing motor carriers (16). Still, some state and local laws have survived preemption scrutiny to limit indoor (and even some outdoor) smoking (1), ban tobacco sales to minors (13), and raise the minimum age of tobacco sales and possession to 21 years (as in Hawaii in 2015) (17).

Preemption themes are also evinced in laws addressing obesity and related chronic conditions (eg, heart disease, diabetes, cancer) (18). Cities pioneered the concept of menu labeling of calorie and other nutritional information (19). New York City enacted the first law requiring calorie disclosure on restaurant menus in 2006 (20). When this initial law was challenged, a court ruled that it was preempted by the federal Nutrition Labeling and Education Act of 1990 (NLEA), which regulates voluntary nutritional claims by restaurants (21). Undeterred, the city revised the law to require all restaurants with 15 or more locations, even those who did not make voluntary nutritional claims, to provide factual calorie information. The revised law survived a second preemption challenge because the NLEA does not regulate restaurants providing basic nutritional information (22).

As more localities caught on and research demonstrated the potential utility of menu labeling to lower calorie intakes, the restaurant industry sought to preempt varied state and local labeling laws through the federal Patient Protection and Affordable Care Act (ACA) in 2010 (23). ACA requires uniform calorie disclosures by certain chain restaurants and preempts more stringent state or local laws (19). It exemplifies the 2 sides of preemption. While federal law mandates menu labeling in states and localities that might never pass similar laws, it also stymies creative and varied local regulations by preempting stricter subnational laws. State and local governments can still require posting of additional nutritional information beyond calories (eg, trans fat, cholesterol, sodium) but only by restaurants, theaters, bowling alleys, or other food-serving establishments not covered by ACA (19).

Sometimes the effects of preemption on public health innovations are less direct. When new legislation is being considered at the state or local level, industry’s mere assertions that the desired law or regulation could be preempted may keep a bill from ever being introduced or enacted (1). State and particularly local governments may lack the resources to engage in lengthy and uncertain legal battles over preemption issues or related threats by higher levels of government. City officials in Tempe, Arizona, recently abandoned plans to require employers to provide paid sick and safe time when Arizona’s state legislature threatened to withhold all of its state-based funding if the city enacted its plan (24).

## Navigating Preemption to Protect the Public’s Health

Though subject to potential pitfalls, successful navigation of the legal system to promote the public’s health at state and local levels is possible, consistent with the following guidance:

**Stand firm on legal grounds.** Attempts to preempt areas of public health law must rely on sufficient authority. If public health legal innovation is skirted by higher-level laws lacking the required authority, challenge the higher laws directly. When Ohio passed legislation designed to preempt the City of Cleveland’s existing law banning the use of trans fat in city restaurants, Cleveland sued. Because of the manner in which the state law was enacted, the court held that the state law unconstitutionally stripped Cleveland of its local home rule, resulting in the reinstatement of the city’s ban (25).
**Find the floor of preemption and rise above it**. A standard preemptive technique is to create a floor of regulation under which no lower level law may go. Less appreciated is that state or local public health laws can exceed the minimums without violating preemption in some cases. Federal spending conditions support that persons must be 18 years of age to purchase tobacco products but do not forbid a state or local jurisdiction from exceeding this threshold. California’s legislature recently matched Hawaii’s “age 21” tobacco use law (17).
**Recharacterize the public health objective.** If Congress “occupies the field,” preempting lower-level regulations in one area, state and local governments should consider alternative laws to accomplish similar objectives. As noted above, when the NLEA preempted state or local regulation of restaurants’ voluntary nutritional claims (21), New York City instead required restaurants to provide factual nutritional information that avoided preemption.
**Fill the inevitable gaps.** Inherent limits in the political process result in compromises in virtually every law with preemptive effects. The objective is to find the gaps and fill them with unique efforts designed to advance public health goals without tripping over preemption. Coverage gaps inherent in ACA’s menu-labeling provisions, discussed above, limit them to chain restaurants with 20 or more locations. State and local governments can effectively regulate thousands of restaurants with fewer locations or other establishments serving food. In other cases, legislation may feature “grandfather” clauses that allow existing state or local measures to largely remain in place.
**Reconsider nonregulatory solutions**. A perceived problem with preemption is that it can circumvent or override state or local legal innovations. Sometimes, the actual problem is that state or local officials did not fully consider nonlegal solutions. Other, less public interventions (eg, industry agreements, public health education, targeted media campaigns) may be equally efficacious as legislation or regulation but better insulated from preemptive effects.

The complexities of legal preemption may be daunting, but tactical strategies grounded in basic legal understanding, advance policy planning, and analyses of varied legal options can lead to real solutions that promote efforts to address the impacts of chronic conditions on populations.
